# In chronic spontaneous urticaria, IgE and C‐reactive protein are linked to distinct microRNAs and interleukin‐31

**DOI:** 10.1002/clt2.12290

**Published:** 2023-08-07

**Authors:** Ozge Sevil Karstarli Bakay, Betül Demir, Demet Cicek, Deniz Erol, Zulal Aşçı Toraman, Yunus Gural, Marcus Maurer

**Affiliations:** ^1^ Department of Dermatology Pamukkale University Faculty of Medicine Denizli Turkey; ^2^ Department of Dermatology Firat University Faculty of Medicine Elazig Turkey; ^3^ Department of Medical Genetics Firat University Faculty of Medicine Elazig Turkey; ^4^ Department of Microbiology Firat University Faculty of Medicine Elazig Turkey; ^5^ Division of Statistics Firat University Faculty of Science Elazig Turkey; ^6^ Urticaria Center of Reference and Excellence (UCARE) Institute of Allergology Charité – Universitätsmedizin Berlin, corporate member of Freie Universität Berlin and Humboldt‐Universität zu Berlin Berlin Germany; ^7^ Fraunhofer Institute for Translational Medicine and Pharmacology ITMP, Allergology, and Immunology Berlin Germany

**Keywords:** CRP, IgE, interleukin‐31, micro‐RNA, urticaria

## Abstract

**Background:**

Chronic spontaneous urticaria (CSU) is a common and disabling disease. Assessments of IgE and C‐reactive protein (CRP) are recommended in the diagnostic work‐up, but the role and clinical relevance of these biomarkers are not well characterized. Moreover, it remains unknown if elevated levels of IgE or CRP are linked to CSU microRNA (miRNA) signatures or interleukin 31 (IL‐31).

**Methods:**

We measured IgE and CRP serum levels in 47 CSU patients (and 45 healthy controls) and determined CSU disease activity using the urticaria activity score (UAS7). Expression levels of miR‐155 and miR‐221 were assessed by RT‐PCR, and IL‐31 levels were determined by ELISA.

**Results:**

Total IgE and CRP levels were independently increased in CSU patients. IgE and CRP levels were highest and lowest in patients with high and mild disease activity. IgE levels correlated with miR‐155 levels, whereas CRP levels correlated with miR‐221 levels. miR‐155 and miR‐221 were significantly overexpressed in CSU patients. ROC analyses linked miRNA‐155 and CSU with a sensitivity of 79% and specificity of 87%, and miRNA‐221 and CSU with a sensitivity of 75% and specificity of 91%. High CRP and miR‐221 expression levels were linked to elevated levels of IgG anti‐TPO and IL‐31.

**Conclusion:**

IgE and CRP are useful biomarkers for disease activity in CSU, with distinct miRNA profiles. High CRP and miR‐221 levels may point to autoimmune CSU and a role for IL‐31.

## 1 INTRODUCTION

Chronic spontaneous urticaria (CSU) is a common and disabling inflammatory disease characterized by itchy wheals, angioedema, or both.^1^ These signs and symptoms are caused by the activation and degranulation of skin mast cells and their release of proinflammatory mediators including histamine.^2^ In most patients with CSU, skin mast cells are activated by autoantibodies, either IgE antibodies against autoantigens, in autoallergic CSU, or IgG autoantibodies against IgE or its high‐affinity receptor, FceRI, in autoimmune CSU.^3^ Autoallergic and autoimmune CSU differ in prevalence, disease activity and duration, and response to treatment. Autoimmune CSU is characterized by high disease activity, the presence of comorbid autoimmune diseases, elevated levels of IgG‐anti–thyroid peroxidase, basopenia, eosinopenia, and poor response to antihistamine and omalizumab treatment.^4^ In routine clinical practice, it is important but difficult to assess patients for autoimmune CSU because three tests need to be performed and be positive, that is, the autologous serum skin test (ASST), an immunoassay for IgG anti‐IgE/FceRI, and a basophil activation test.^5^

The most recent version of the international guideline for urticaria^6^ provides recommendations for the diagnostic work‐up of CSU patients. These include the following blood tests, all of which are readily available for use in routine clinical practice: C‐reactive protein (CRP) and/or ESR, a differential blood count, total IgE, and IgG‐anti‐TPO. Some of these blood tests can help to screen CSU patients for the underlying cause of their disease, that is, autoallergy and autoimmunity. For example, elevated blood levels of total IgE, in some studies, were linked to autoallergic CSU, whereas low IgE levels point to autoimmune CSU.^7^ Elevated levels of CRP/ESR have also been suggested to be linked to autoimmune CSU.^8^ To date, however, the clinical profiles of CSU patients with high IgE or elevated CRP remain ill‐defined. Whether these two CSU biomarkers are linked to inflammation‐associated microRNAs (miRNA) or the itch cytokine interleukin‐31 (IL‐31) is currently unknown.

miRNAs, small noncoding RNAs, are believed to be involved in the pathogenesis of several chronic inflammatory skin disorders,^9^ but little is known about their role in CSU.^10^ Two miRNAs, miRNA‐155 (miR‐155) and miRNA‐221 (miR‐221), are of special interest in CSU as they have been implicated in the modulation of mast cell activation, IgE and FceRI, and autoimmunity. miR‐155 is needed for antibody production, B‐cell maturation, differentiation, and immunoglobulin class switching.^11^ miR‐155 has also been reported to downregulate FceRI expression and FcεRI‐mediated mast cell degranulation,^12^, ^13^ and its expression is increased in human skin‐derived mast cells following FcεRI crosslinking with antigen.^14^ CSU patients have not yet been assessed for miR‐155, but patients with atopic dermatitis exhibit increased expression in mast cells at lesional skin sites.^15^ miR‐221 contributes to the regulation of the cell cycle and cytoskeleton of mast cells as well as their degranulation and cytokine production.^16^ miR‐221 has also been linked to several autoimmune disorders, including psoriasis^17^ and rheumatoid arthritis,^18^ but, like miR‐155, has not been studied in CSU.

Interleukin 31 is involved in the pathogenesis of chronic inflammatory skin disorders, including atopic dermatitis, allergic contact dermatitis, and mastocytosis.^19^, ^20^, ^21^, ^22^ It is also believed to contribute to the pathogenesis of CSU, although there is little evidence for this. In a recent study, CSU patients had significantly higher mean serum IL‐31 levels as compared to healthy subjects. Interestingly, CSU patients with elevated ANA titers, a marker of autoimmune CSU, had significantly higher mean serum IL‐31 levels than those who were negative for ANA.^23^ Also, successful omalizumab treatment of patients with CSU is associated with lowering serum IL‐31 levels.^24^ Whether IL‐31, in CSU, is linked to IgE or CRP is currently unknown.

To address these gaps of knowledge, we investigated CSU patients for total IgE and CRP levels and their links to clinical features. We then assessed miR‐155 and miR‐221 levels and their association with IgE and CRP levels. Finally, we explored interleukin‐31 levels in CSU and how they are linked to IgE, CRP, and miRNAs.

## 2 METHODS

### 2.1 Study subjects and conduct

This study was conducted at the Dermatology outpatient clinic of Firat University Faculty of Medicine with a total of 100 adult participants, that is, 50 patients with CSU and 50 healthy individuals, and the sociodemographic characteristics of all the participants were recorded. Each patient underwent a detailed dermatological examination and completed the urticaria activity score (UAS). Laboratory parameters, that is, CRP, IgG anti‐thyroid peroxidase (anti‐TPO), and total IgE, were determined by enzyme‐linked immunosorbent assay (ELISA). The upper limit of normal for total IgE was 100 IU/ml. The study was initiated after obtaining the approval of the local ethics committee (Date: 29/09/2016, no: 02). All participants provided written informed consent.

### 2.2 Assessment of disease activity by the use of the urticaria activity score

The disease activity of CSU patients was assessed with the urticaria activity score (UAS) as recommended using the EAACI/GA^2^LEN/EuroGuiDerm/APAAACI guideline for urticaria.^6^ Patients recorded daily, for 7 days, the number of wheals (no wheals = 0 points; <20 wheals = 1 point; 20–50 wheals = 2 points; >50 wheals = 3 points) and the intensity of itch (no itch = 0 points; mild itch = 1 point; moderate itch = 2 points; severe itch = 3 points). The weekly UAS (UAS7) was calculated by adding the UAS values of 7 consecutive days, with a minimum score of 0 and a maximum score of 42. Patients with UAS7 values of 1–15, 16–27, and 28–42 were considered to have minimal/mild, moderate, and severe disease activity, respectively.^25^

### 2.3 RT‐PCR analysis of plasma samples

Total RNA was isolated from serum samples (200 μL per sample) of 50 CSU patients and 50 controls using a miRNeasy Serum/Plasma Kit (Qiagen, Hilden, Germany) according to the manufacturer's instructions. Qiagen miScript Reverse Transcription (RT) Kit II (Hilden, Germany) was used for cDNA extraction. The cDNAs were amplified using the Qiagen miScript PreAMP PCR kit (Hilden, Germany). Following the cDNA extraction, the real‐time polymerase chain reaction (RT‐PCR) stage began to analyze the expression levels of miR‐155 and miR‐221. The reaction components for RT‐PCR were prepared using the Qiagen miScript SYBR Green PCR kit. SNORD61 was used for the normalization of RT‐PCR. RT‐PCR Rotor‐Gene Q (Qiagen) was used to identify SNORD61 expression levels as a reference along with the hsa‐miR‐155‐3p and hsa‐miR‐221‐3p miRNA primers.

### 2.4 Calculation of fold changes by 2^−ΔΔCt^ analysis

Changes in miR‐155 and miR‐221 gene expression were calculated. First, the delta CT values of SNORD61 were calculated considering it as the reference miRNA. The Ct values provided by the Real‐Time analysis were entered into the Excel program, and the statistical 2^−ΔΔCt^ analyzed of all miRNAs using the GeneGlobe Data Analysis Center (Qiagen) online analysis program.

### 2.5 IL‐31 analysis

Serum samples obtained by centrifugation were stored at −80°C and concentrations of IL‐31 in serum samples were measured in 47 CSU patients and 45 control subjects by ELISA using commercially available kits (Elabscience Biotechnology Co. Ltd).

### 2.6 Statistical analysis

Statistical analysis was performed using the Statistical Package for Social Sciences for Windows (SPSS) 22.0. The results of categorical measurements were expressed as numbers and percentages. The chi‐square test was used for the analysis of two categorical variables. Normally distributed numeric measurements were presented as mean ± standard deviation, and non‐normally distributed numeric measurements were given as mean ± standard deviation, median (percentiles 25th–75th). The Mann‐Whitney *U* test was used to compare non‐normally distributed numerical data between two groups and the Kruskal‐Wallis test was used to compare the means among three or more groups (Significance values have been adjusted by the Bonferroni correction for multiple tests). Independent sample *t*‐test was used to compare normally distributed data between two groups, whereas one‐way ANOVA was used to compare the means among three or more groups. Spearman and Pearson correlation tests were used for correlation analysis. A *p*‐value of <0.05 was considered statistically significant.

## 3 RESULTS

In the patient group (32 females), the mean age was 35.6 ± 6.8; and in the control group (34 females), the mean age was 33.5 ± 9.9 years. Based on their UAS7 values, 6 (12.8%), 20 (42.6%), and 21 (44.7%) patients had minimal/mild, moderate, and severe disease activity, respectively.

### 3.1 Blood levels of total IgE and CRP are independently increased and linked with disease activity in patients with CSU

**FIGURE 1:**
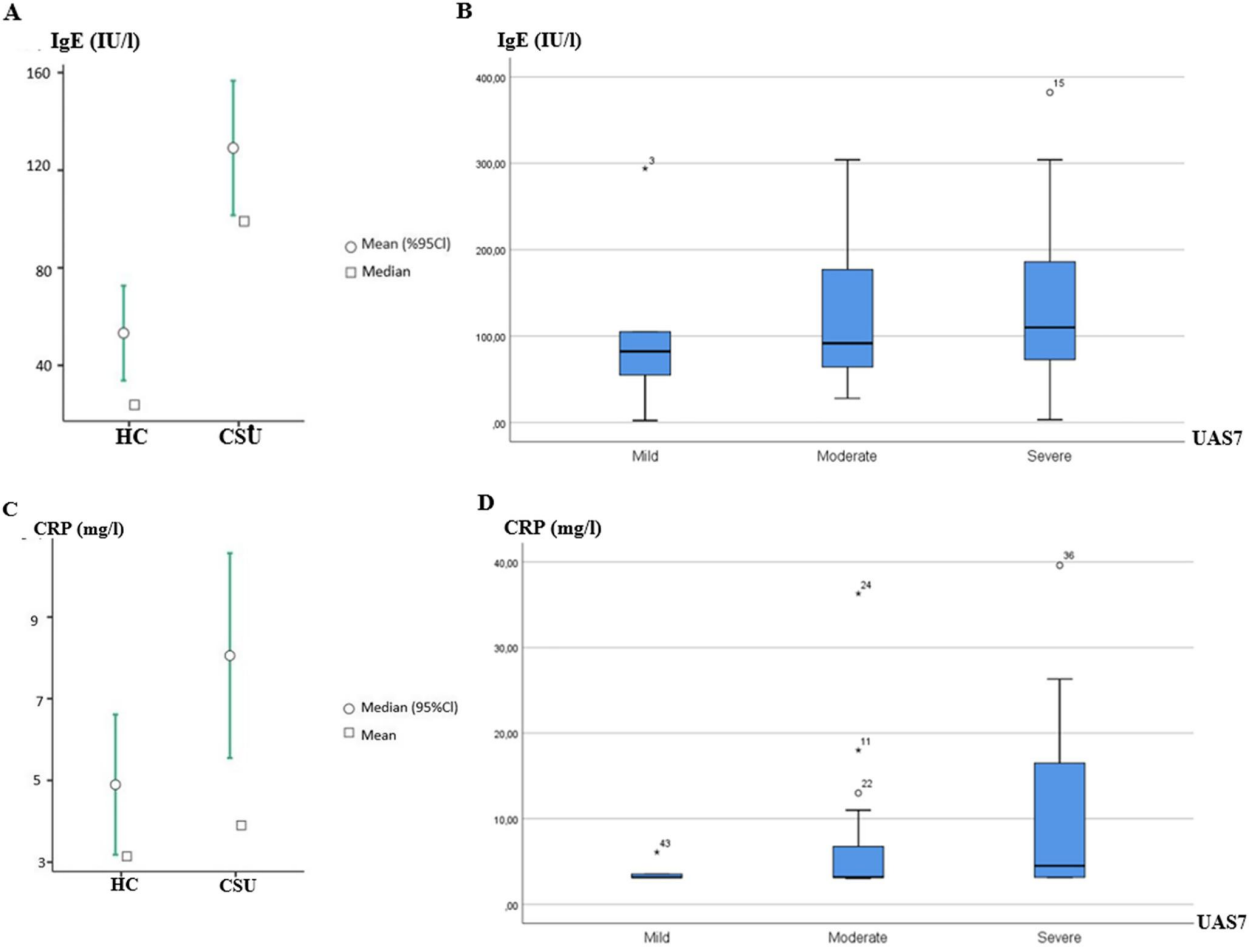
IgE and CRP levels increased independently and were linked to disease activity in CSU patients. (A) Total IgE levels were significantly higher in CSU patients (129 ± 14 IU/L) than in HCs (53 ± 9 IU/L, *p* < 0.01). (B) IgE levels were highest (138 ± 105 IU/L) in patients with high disease activity, intermediate in patients with moderate disease activity (127 ± 89 IU/L), and lowest (104 ± 100 IU/L) in patients with mild disease activity. (C) CRP levels were higher in CSU patients (8.1 ± 1.3 mg/L) than in HCs (4.8 ± 0.8 mg/L, *p* < 0.05). (D) CRP levels were highest (10.3 ± 10.2 mg/L) in patients with high disease activity, intermediate in patients with moderate disease activity (6.9 ± 8.0 mg/L), and lowest in patients with mild disease activity (3.7 ± 1.2 mg/L). CRP, C‐reactive protein; CSU, chronic spontaneous urticaria; HC, heathy controls, disease activity assessed by UAS7; UAS, urticaria activity score.

Mean total IgE levels were significantly higher in CSU patients (129 ± 14 IU/L) as compared to healthy controls (53 ± 9 IU/L, *p* < 0.01; Figure 1A). More than half of the CSU patients, 24 of 47 (51.4%), had elevated total IgE levels as compared to 8 of 45 healthy controls (17.7%, *p* < 0.05). Across all CSU patients, IgE levels were highest [138 ± 105 IU/L, median = 110 IU/L (65.0, 197.5 IU/L)] in patients with high disease activity, followed by moderate disease activity [127 ± 89 IU/L, median = 91.7 IU/L (60.62, 187.0 IU/L)], and they were lowest [104 ± 100 IU/L, median = 82.3 IU/L (41.8, 152.2 IU/L)] in patients with mild disease activity (Figure 1B), as assessed by UAS7.

CSU patients also had higher CRP levels than healthy controls, that is, 8.1 ± 1.3 and 4.8 ± 0.8 mg/L, respectively (*p* < 0.05; Figure 1C), and higher rates of elevated CRP (36.2% vs. 13.3%). CRP levels were highest [10.3 ± 10.2 mg/L, median = 4.5 mg/L (3.15, 16.55 mg/L)] in patients with high disease activity, lowest in patients with mild disease activity [3.7 ± 1.2 mg/L, median = 3.1 mg/L (3.01, 3.35 mg/L)] and intermediate in patients with moderate disease activity [6.9 ± 8.0 mg/L, median = 3.2 mg/L (3.14, 6.7 mg/L); Figure 1D]. There was no correlation between IgE and CRP levels in CSU patients.

### 3.2 IgE and CRP are independently linked to miR‐155 and miR‐221, respectively, both of which are overexpressed in CSU

**FIGURE 2:**
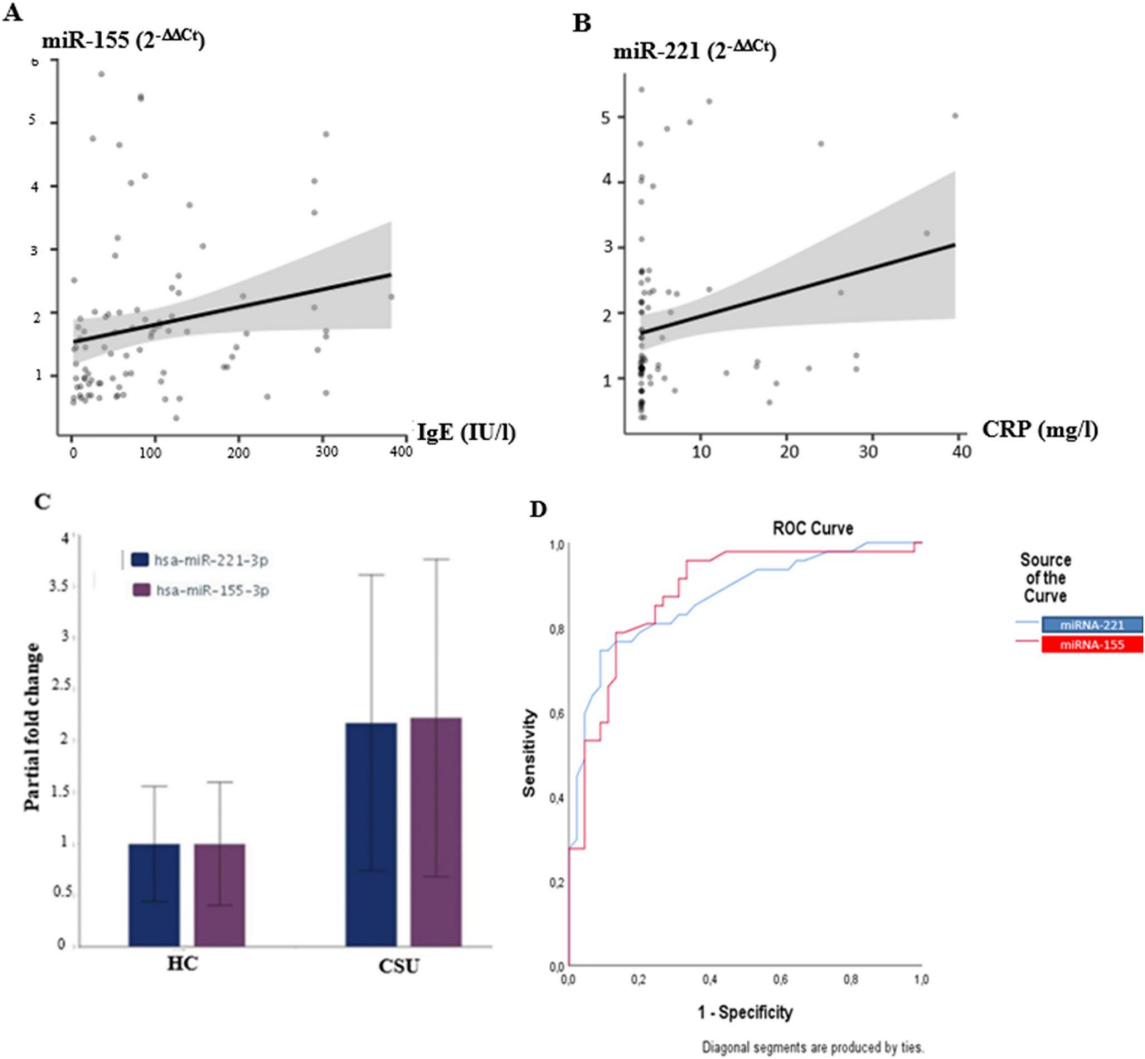
miR‐155 and miR‐221 are upregulated in CSU and linked to total IgE and CRP, respectively. (A) IgE levels correlated with miR‐155 levels in all study participants (*r* = 0.28, *p* < 0.05). (B) CRP levels correlated with miR‐155 levels in all study participants (*r* = 0.25, *p* < 0.05). (C) miR‐221 and miR‐155 were correlated and significantly higher in CSU patients than in HCs (fold change>2.2, *p* < 0.01). (D) ROC analyses linked miR‐155 and CSU with a sensitivity of 79% and specificity of 87%, and miR‐221 and CSU with a sensitivity of 75% and specificity of 91%. CRP, C‐reactive protein; CSU, chronic spontaneous urticaria; HC, heathy controls.

Across all study subjects, IgE levels, but not CRP levels, were correlated with levels of miR‐155 (*r* = 0.28, *p* < 0.05; Figure 2A), whereas levels of CRP, but not IgE, were correlated with levels of miR‐221 (*r* = 0.25, *p* < 0.05; Figure 2B). In CSU, the expression levels of miR‐221 and miR‐155 were correlated and significantly higher as compared to the control group (>2.2‐fold, *p* < 0.01; Figure 2C). ROC analyses linked miR‐155 and CSU with a sensitivity of 79% and specificity of 87%, and miR‐221 and CSU with a sensitivity of 75% and specificity of 91% (Figure 2D).

### 3.3 CRP and miR‐221 expression are linked to features of autoimmune CSU and interleukin‐31

**FIGURE 3:**
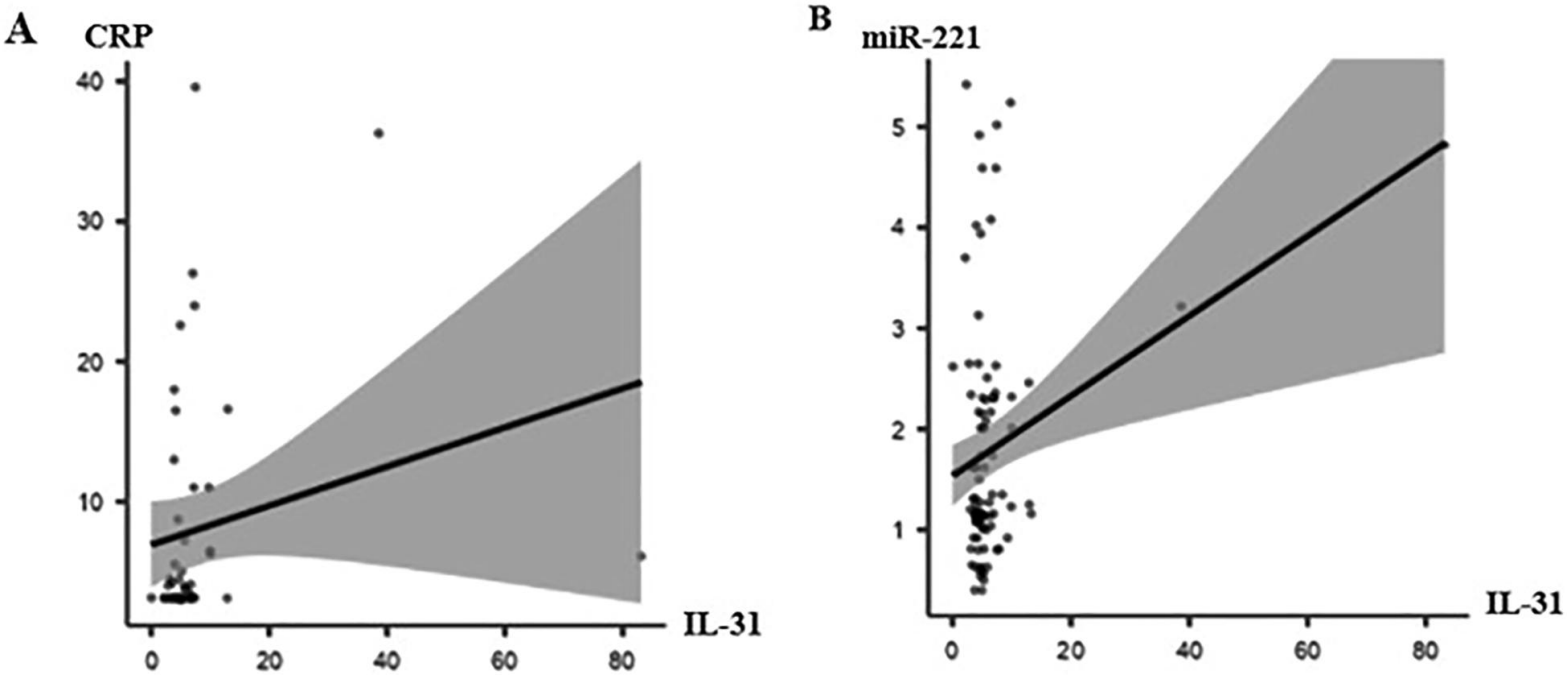
CRP and miR‐221 expression are linked to IL‐31. A. In CSU patients, there was a positive correlation between CRP levels and blood levels of IL‐31 (*r* = 0.36, *p* < 0.05). B. IL‐31 and miR‐221 expression levels showed a weak but significant correlation (*r* = 0.29, *p* < 0.05) in the entire study group. CRP, C‐reactive protein; CSU, chronic spontaneous urticaria.

In CSU patients, levels of CRP correlated with those of anti‐TPO (*r* = 0.33, *p* < 0.05), and anti‐TPO‐positive patients had higher CRP levels as compared to anti‐TPO‐negative patients [16.5 ± 15.9 mg/L, median = 8.74 mg/L (3.93, 32.95 mg/L) versus 7.0 ± 7.2 mg/L, median = 3.37 mg/L (3.14, 6.69 mg/L), *p* < 0.05]. High CRP was also linked to elevated blood levels of IL‐31 (*r* = 0.36 *p* < 0.05) (Figure 3A). The mean IL‐31 level in patients in the high CRP group was markedly higher than the mean IL‐31 level in the low CRP group [12.8 ± 19.3 pg/mL, median = 7.14 pg/mL (4.43, 10.0 pg/mL) versus 5.0 ± 2.3 pg/mL, median = 5.06 pg/mL (3.41, 6.46 pg/mL), *p* = 0.01] (Table 1).

**TABLE 1:** Laboratory findings (a) and clinical characteristics (b) in CSU patients with low and high CRP levels.

(a) Laboratory findings	Low CRP	High CRP	p value
	Mean ± SD	Mean ± SD	
	Median, percentiles^a^	Median, percentiles^a^	
miR‐221 (2^−ΔΔCT^)	2.42 ± 1.15	2.61 ± 1.59	0.651
miR‐155 (2^−ΔΔCT^)	2.48 ± 1.43	2.35 ± 1.19	0.756
IL‐31 (pg/dL)^a^	5.02 ± 2.28	12.77 ± 19.25	**0.01**
	5.06 (3.41, 6.46)	7.14 (4.43, 10.0)	
Total IgE (IU/L)^a^	125.4 ± 93.8	135.1 ± 102.9	0.948
	95.8 (72.0, 171.5)	112.5 (54.25, 229.25)	

(b) Clinical characteristic		Low CRP *n* (%)	High CRP *n* (%)	p value
Duration	<1 year	19 (65.5)	7 (38.9)	**<0.05**
	>1 year	10 (34.5)	11 (61.1)	
Atopic status	Yes	1 (3.4)	5 (27.8)	**<0.05**
	No	28 (96.6)	13 (72.2)	
Angioedema	Yes	10 (34.5)	9 (50)	>0.05
	No	19 (65.5)	9 (50)	
ASST	Negative	23 (79.3)	15 (83.3)	>0.05
	Positive	6 (20.7)	3 (16.7)	
UAS7	Mild	5 (17.2)	1 (5.6)	>0.05
	Moderate	13 (44.8)	7 (38.9)	
	Severe	11 (37.9)	10 (55.6)	

*Note*: Low CRP <5 mg/L; high CRP >5 mg/L. Significant results indicating *p*‐values below 0.05 are reported in bold.

Abbreviations: ASST, autologous serum skin test; CRP, C‐reactive protein; IgE, immunoglobulin E; IL‐31, interleukin 31; miR, microRNA; UAS, urticaria activity score.

^a^ Results of non‐parametric tests are shown as mean ± standard deviation and median and 25th and 75th percentiles.

Expression levels of miR‐221 were higher in anti‐TPO‐positive patients than in negative patients (3.5 ± 1.4 vs. 2.4 ± 1.3, *p* < 0.05). MiR‐221 expression levels were highest (2.7 ± 1.4) in patients with high disease activity, lowest in patients with mild disease activity (2.1 ± 1.2), and intermediate in patients with moderate disease activity (2.4 ± 1.3). Across the entire study population, expression levels of miR‐221 were significantly correlated with levels of interleukin‐31 (*r* = 0.29, *p* < 0.05), albeit weakly (Figure 3B). Both, miR‐221 and CRP levels, were significantly higher in patients who had their CSU for longer than 1 year (*p* < 0.01, *p* < 0.05, respectively).

## 4 DISCUSSION

This study identified several novel features of CSU, most importantly that total IgE and CRP are independently increased and linked to disease activity, that IgE and CRP are independently linked to miR‐155 and miR‐221, respectively, and that CRP and miR‐221 expression are linked to features of autoimmune CSU and interleukin‐31.

It is well known that CRP and total IgE levels are elevated on average in CSU, and it has also been suggested that they are potential biomarkers for disease activity.^7^, ^8^, ^26^, ^27^ Although the rates reported in studies vary, IgE is elevated in approximately half of the CSU patients^7^, ^28^ and CRP in about one‐third of CSU patients.^8^ In our study, IgE elevation was found in 51.4% of the patients and CRP in 36.2%, confirming previous reports.

Total IgE has been the subject of numerous studies in CSU over the past years, but there are few studies on its association with disease activity, with contradictory results.^29^, ^30^, ^31^ Some studies found IgE levels to be correlated with disease activity,^30^, ^31^ whereas others did not.^27^, ^29^ The reasons for this may include differences in study populations, including rates of comorbid atopic diseases.

Most studies on CRP levels in CSU found them to be linked with disease activity,^8^, ^27^, ^32^, ^33^, ^34^ but two did not.^26^, ^29^ In our study, total IgE and CRP levels were highest in severe disease and lowest in mild disease activity. However, they were not correlated with each other, which supports their association with different CSU endotypes, that is, autoallergic and autoimmune CSU.^7^, ^8^ IgE antibodies against interleukin (IL)‐24,^35^ thyroid peroxidase,^36^, ^37^ double‐stranded DNA,^38^ tissue factor,^39^ and thyroglobulin^40^ in CSU patients are seen as drivers of the pathogenesis of autoallergic CSU. Many but not all studies have linked the presence of IgE autoantibodies to elevated total IgE levels in CSU patients.^37^, ^41^ Clinical features such as a higher frequency of atopy^37^ and a rapid response to omalizumab^42^ have been reported in CSU patients with high IgE levels. In a recent study, similar to our results, total IgE and CRP values were also not correlated with each other.^27^

Remarkably, CSU patients had higher levels of miR‐155 and miR‐221. This has not previously been reported but does not come completely unexpected. miR‐155 and miR‐221 have been shown to have effects on mast cell functions.^14^, ^16^, ^43^, ^44^ Furthermore, overexpression of miR‐155 has been detected in mast cell‐driven diseases such as atopic dermatitis,^15^, ^45^ allergic contact dermatitis,^46^ asthma,^47^ and allergic rhinitis,^48^ while overexpression of miR‐221 has been detected in chronic inflammatory diseases such as psoriasis,^17^ psoriatic arthritis,^49^ rheumatoid arthritis,^18^ and ankylosing spondylitis.^50^

Even more remarkably, miR‐155 was linked to IgE and miR‐221 to CRP. Why IgE and miR‐155 are associated is currently unclear, but they are also linked in atopic dermatitis (AD), where total IgE levels tend to be markedly higher than in CSU.^7^ In a previous study, patients with atopic dermatitis had 4.6‐fold higher miR‐155,^15^ as compared to 2.2‐fold elevated levels in our CSU patients. Interestingly, miR‐155 suppresses CTLA4, an inhibitory molecule of T cell response, in activated T cells.^15^ In animal models, CTLA‐4 blockade has been shown to maintain or increase allergic responses and inflammation with increased IgE levels.^51^ Furthermore, miR‐155 was upregulated in type 2 native lymphoid cells in response to IL‐33,^52^ which can be increased in CSU^53^, ^54^ and causes elevated serum IgE.^55^ Further studies are needed to better characterize the relationship between miR‐155, IgE, and IL‐33 in the pathogenesis of CSU.

Of note, miR‐155 is encoded within a region known as the B cell integration cluster (Bic) gene located on chromosome 21q21,^56^ which has been linked with IgE‐mediated diseases.^57^ In fact, miR‐155 is implicated in the pathogenesis of allergic diseases such as asthma,^47^ allergic rhinitis,^58^ and atopic dermatitis,^15^ and many studies show that it plays an important role in IgE‐mediated immune responses.^14^, ^43^ Also, miR‐155 expression was increased in human skin‐derived mast cells and mouse bone marrow‐derived mast cells following FcεRI crosslinking with IgE antigen.^14^ On the other hand, miR‐155 increased IgE‐dependent cytokine production by targeting the suppressor of cytokine signaling 1.^43^ It was shown that miR‐155 specifically targets the FcεRI signaling pathway leading to prostaglandin biosynthesis and cytokine production but not the leukotriene production or degranulation pathways.^14^ These results suggest positive feedback between IgE‐mediated cytokine release and miR‐155 expression. It has been reported that miR‐155 expression is higher in patients with allergic rhinitis than in those with non‐allergic rhinitis. In the same study, miR‐155 expression was found to be higher in patients with a positive skin prick test, which detects type I reactions related to allergens, compared with patients with negative prick test.^58^ In our study, miR‐155 was overexpressed in CSU patients and had a specificity of 87% by ROC analysis. Taken together, these results suggest that miR‐155 is a biomarker for CSU, especially for autoallergic CSU.

CRP levels have previously been reported to be higher in autoimmune CSU.^8^, ^32^ Our finding of a correlation between CRP and anti‐TPO, a marker of autoimmune CSU, supports this notion. As a remarkable and new finding, one important parameter we found to be correlated with CRP was miR‐221. miR‐221 has been studied in autoimmune diseases such as psoriasis,^17^ psoriatic arthritis,^49^ and rheumatoid arthritis^54^ and was suggested to be a biomarker for disease activity, early diagnosis, or response to treatment. For example, miR‐221 expression was increased in psoriasis and correlated with disease activity and inflammatory cytokine levels (tumor necrosis factor‐α, IL‐17A, and IL‐22).^17^ Also, miR‐221 expression was increased in patients with psoriatic arthritis, and low miR‐221 levels were associated with poor treatment response.^49^ In rheumatoid arthritis, miR‐221 was higher in patients with high disease activity, and the reduction of inflammatory cytokines (TNFa, IL‐6, and IL‐1β) by inhibition of miR‐221 suggests that it may be a target for treatment.^18^, ^59^ The fact that miR‐221 is more strongly stimulated by non‐IgE antibodies in previous studies suggests that the association between miR‐221 and anti‐TPO or CRP is not accidental and indicates that miR‐221 is a biomarker for autoimmune CSU.

We also observed that both CRP and miR‐221 are linked to IL‐31. IL‐31, a member of the IL‐6 cytokine family, is a pruritic proinflammatory cytokine.^60^ Not surprisingly, IL‐31 levels are elevated in pruritic diseases such as atopic dermatitis,^19^ prurigo nodularis,^61^ lichen planus,^62^ and uremic pruritus.^63^ Furthermore, elevated IL‐31 levels have been reported in autoimmune diseases such as psoriasis,^64^ systemic lupus erythematosus,^65^ dermatomyositis,^66^ bullous pemphigoid,^67^ and subtypes of pemphigus including the non‐pruritic subtype pemphigus vegetans.^68^

IL‐31 levels were previously reported to be elevated in CSU, but its role in the pathogenesis has not been clarified yet.^20^, ^23^, ^69^ The decrease in IL‐31 levels after omalizumab treatment in CSU patients suggests that IL‐31 is a pathogenic driver of CSU.^24^ In line with our results, IL‐31 was not associated with disease activity as measured by UAS7 in previous studies,^23^, ^24^, ^54^ although results regarding the correlation with itch scores are conflicting.^23^, ^54^ The detection of higher IL‐31 levels in patients with high ANA titers^23^ prompts us to consider whether IL‐31 contributes to the pathogenesis of autoimmune CSU, although there is insufficient evidence for this. IL‐31 levels were higher in CSU patients with thyroid autoimmunity (assessed by anti‐TPO and/or anti‐TG) than in patients without thyroid autoimmunity, but the difference was not statistically significant.^23^ In previous studies, there was no difference in IL‐31 plasma levels in ASST‐positive or negative CSU patients,^69^, ^70^ and IL‐31 was not linked to CRP.^54^ In contrast, in our study, IL‐31 was linked to miR‐221 and CRP. The mean IL‐31 level in patients in the high CRP group was higher than the mean IL‐31 level in the low CRP group. A previous CSU study suggested that IL‐31 is not the primary mediator of pruritus, as some patients with high disease activity had undetectable levels of IL‐31, and the highest IL‐31 level was lower than what is required to induce itch sensation.^24^ The fact that IL‐31 is not only elevated in pruritic skin diseases but also in autoimmune diseases suggests that it plays an important role in inflammatory responses and autoimmunity beyond itch.

Our study has two limitations: (i) a relatively small number of patients and (ii) the absence of post‐treatment levels of the biomarkers assessed. However, the strength of the study is that it is a prospective study in which the clinical and laboratory findings of the patients were evaluated comprehensively.

## 5 CONCLUSİON

Total IgE and CRP appear to be drivers in different subtypes of CSU and may be biomarkers of disease activity. Upregulation of miR‐155 and miR‐221 with high specificity in CSU patients makes their role in CSU pathogenesis worth investigating. The association of miR‐221 with CRP, anti‐TPO, IL‐31, and disease activity indicates that it is a potent biomarker for CSU, especially for autoimmune CSU. Further studies should explore the role of miRNAs and interleukin‐31 signatures in CSU.

## AUTHOR CONTRIBUTIONS

Ozge Sevil Karstarli Bakay and Betül Demir designed the study. Ozge Sevil Karstarli Bakay, Betül Demir, and Demet Cicek coordinated the study. Deniz Erol and Zulal Aşçı Toraman established and performed the laboratory tests. Demet Cicek and Yunus Gural performed statistical analyses. Ozge Sevil Karstarli Bakay and Marcus Maurer were involved in data interpretation. Ozge Sevil Karstarli Bakay and Marcus Maurer drafted the manuscript. All authors proof‐read and approved the final version of the manuscript.

## CONFLICT OF INTEREST STATEMENT

None, in relation to this work. Outside of it, MM is or recently was a speaker and/or advisor for and/or has received research funding from Allakos, Amgen, Aralez, ArgenX, AstraZeneca, Celldex, Centogene, CSL Behring, FAES, Genentech, GIInnovation, GSK, Innate Pharma, Kyowa Kirin, Leo Pharma, Lilly, Menarini, Moxie, Novartis, Pfizer, Roche, Sanofi/Regeneron, Third Harmonic Bio, UCB, and Uriach.

## Data Availability

Data can be requested by the corresponding authors.
